# Squamate reptiles may have compensated for the lack of γδTCR with a duplication of the TRB locus

**DOI:** 10.3389/fimmu.2024.1524471

**Published:** 2025-01-09

**Authors:** Jordan M. Sampson, Kimberly A. Morrissey, Kieran J. Mikolajova, Kourtney M. Zimmerly, Neil J. Gemmell, Michael G. Gardner, Terry Bertozzi, Robert D. Miller

**Affiliations:** ^1^ Center for Evolutionary and Theoretical Immunology, Department of Biology, University of New Mexico, Albuquerque, NM, United States; ^2^ Department of Anatomy, University of Otago, Dunedin, New Zealand; ^3^ College of Science and Engineering, Flinders University, Adelaide, SA, Australia; ^4^ South Australian Museum, Adelaide, SA, Australia; ^5^ School of Biological Sciences, The University of Adelaide, Adelaide, SA, Australia

**Keywords:** squamate, T cells, comparative immunology, gene loss, gene duplication

## Abstract

Squamate reptiles are amongst the most successful terrestrial vertebrate lineages, with over 10,000 species across a broad range of ecosystems. Despite their success, squamates are also amongst the least studied lineages immunologically. Recently, a universal lack of γδ T cells in squamates due to deletions of the genes encoding the T cell receptor (TCR) γ and δ chains was discovered. Here, we begin to address how the loss of γδ T cells may have impacted the evolution of the squamate immune system. Using the skink *Tiliqua rugosa*, we found that squamates have not significantly increased the complexity of conventional T cell receptor beta (TCRβ or *TRB*) chain V regions compared to that of the nearest living squamate relative, the tuatara, *Sphenodon punctatus* or other amniotes. Our analyses include a putative new TCR locus. This novel locus contains V, D, and J gene segments that undergo V(D)J recombination, albeit with a limited number of gene segments in most squamate species. Based on conserved residues, the predicted protein chain would be expected to form a heterodimer with TCRα. This new TCR locus appears to be derived from an ancient duplication of the *TRB* locus and is homologous to the recently described T cell receptor epsilon (*TRE*). *TRE* is absent from the genomes of the tuatara and all Archosaurs examined and appears squamate specific.

## Introduction

The term reptile describes a broad range of species across Sauropsida, the vertebrate clade that includes both the Archelosauria (birds, crocodilians, and turtles) and the Lepidosauria (1, 2). These two lineages diverged between 265-280 million years ago (MYA) (2, 3). The Lepidosauria contains two ancient lineages, the Rhynchocephalia with a single living species, the tuatara *Sphenodon punctatus*, and the Squamata, which are the lizards, snakes, and amphisbaenians (2, 4, 5). Squamata includes more than 10,000 species that occupy a broad range of environmental niches (4, 6–8).

Despite their evolutionary success, reptiles are arguably the least immunologically studied group of vertebrates (9–12). This is unfortunate as squamate reptiles provide many potential model species given their varying life-history traits including viviparity vs. oviparity, sexual reproduction vs. parthenogenesis, and adaptation to a wide range of ecosystems. Nonetheless, there remains comparatively few published squamate immune system studies (9–12).

With few exceptions, all jawed vertebrate immune systems have three distinct lineages of lymphocytes that are clonally unique due to somatic recombination of their antigen receptor genes (13). These receptors are the T cell receptors (TCR) expressed by αβ and γδ T cells and the immunoglobulins (Ig) expressed by B cells (13–15). Squamates lack γδ T cells due to major genomic deletions of the genes encoding the γδTCR chains (16). These deletions occurred after the Lepidosauria-Rhynchocephalia split approximately 260 MYA and appear to be squamate specific as *S. punctatus* has the genes encoding the TCR γ and δ chains (*TRG* and *TRD*) (5, 16, 17).

Here, we examine evidence for possible compensation of the loss of γδ T cells in squamates by investigating the complexity of the remaining TCR loci in a model species, the skink *Tiliqua rugosa.* This analysis includes investigating a potential new TCR chain, recently identified as TCRϵ (*TRE*), that appears to be squamate specific (18). We also provide evidence that this novel TCR is likely derived from a duplication of the TCRβ (*TRB*) locus.

## Materials and methods

### Animals

The *T. rugosa* spleen transcriptome data was generated from two animals, one from Western Australia and one from South Australia described previously in Morrissey et al. (16).

### Genome annotation

The *T. rugosa* genome is being assembled and annotated as part of the Bioplatforms Australia - Australian Amphibian and Reptile Genomics Initiative (https://ausargenomics.com/). The animal used was the same individual, SAMAR71619 (South Australian Museum), used for one of the splenic transcriptomes. Briefly, Verkko (19) was used to generate a hybrid assembly of PacBio HiFi (https://data.bioplatforms.com/ausarg-pacbio-hifi/bpa-ausarg-pacbio-hifi-350719-da052873) and nanopore ultralong reads (https://data.bioplatforms.com/ausarg-ont-promethion/bpa-ausarg-ont-promethion-350780-pag18329), incorporating HiC reads (https://data.bioplatforms.com/ausarg-hi-c/bpa-ausarg-hi-c-350781-hcn7wdrxy) for extended phasing. The resulting pseudohaplotype assemblies and the unassigned contigs were scaffolded separately and together using YaHS (20). HiC contact maps were generated with PretextMap v0.1.90 (https://github.com/sanger-tol/PretextMap) and both haplotypes evaluated simultaneously for misjoins, haplotype switches and other assembly errors with PretextView v0.2.5 (https://github.com/sanger-tol/PretextView) as outlined in https://github.com/Nadolina/Rapid-curation-2.0. Each manually curated pseudohaplotype consists of 16 chromosome sized scaffolds and several unplaced contigs with an average haploid genome size of 1.69G.

Chromosomes containing *TRB* sequences were identified by BLASTn using putative variable (V) and constant (C) gene sequences from the transcriptome analyses (see below). The *T. rugosa* chromosome(s) containing *TRB* was chromosome 2 in both pseudohaplotypes. The *S. punctatus* genome assembly (ASM311381v1, GenBank accession number GCA_003113815.1) was also searched to identify scaffold(s) containing the *TRB* locus. GenBank *TRB* sequences from the chicken, *Gallus gallus*, were used to search the *S. punctatus* whole-genome assembly (accession number EF554755.1). The scaffold containing the *S. punctatus TRB* was scaffold QEPC01009940.1 (https://www.ncbi.nlm.nih.gov/).

Chromosomes containing *TRE* sequences in *T. rugosa* were identified by BLASTn using V and C gene sequences identified from the green anole (*Anolis carolinensis*), originally identified by Gambon-Deza (18) (accession number GCA_035594765.1, NC_085841.1). The *T. rugosa* chromosome(s) containing *TRE* was chromosome 1 in both pseudohaplotypes. The *S. punctatus* genome assembly (ASM311381v1, accession number GCA_003113815.1) was also searched to identify scaffold(s) containing either *TRE* or the *TRE* flanking genes (accession number QEPC01002436.1). Flanking genes were also identified in *A. carolinensis* (accession number GCA_035594765.1, NC_085841.1) and the American alligator (*Alligator mississippiensis*) (accession number GCA_030867095.1,NC_081825.1).

### Transcriptome analysis

Previously published sequences were used to identify the *TRB* transcripts in *T. rugosa* (21). The *TRBC* region identified was used to identify transcripts in a previously published 454 transcriptome dataset (22). The outputs were analyzed for V regions and C regions based on conserved motifs. Identified partial sequences were then used to screen for full length sequences containing V or C regions. Sequences identified were then used to search the PacBio Isoseq transcriptomes with BLASTn in a local database, using the same process described above. Transmembrane regions were identified with DeepTMHMM-2.0 (23). The *T. rugosa TRB* sequences were previously deposited under GenBank accession numbers OL311598-OL311653 (https://www.ncbi.nlm.nih.gov/). The *S. punctatus* transcriptome assembly (GGNQ00000000.1) was searched using similar methods. GenBank accession numbers of all *TRB* sequences identified in the *S. punctatus* transcriptome are found in **Supplementary Table 1**.

To identify transcripts for *TRE*, the *TREC* region identified in the *T. rugosa* genome was used to analyze the same *T. rugosa* PacBio transcriptome (see above). Transcripts were then utilized to screen for sequences containing full-length V or C regions. Transmembrane regions were identified with DeepTMHMM-2.0 (23).

### Annotation and characterization

Non-TCR gene models were predicted using GenSAS with references from non-mammalian vertebrates (24). BLAST was then used on all predicted coding sequences against the GenBank database. Genomic V, D, and J sequences were identified by recombination signal sequences (RSS) or comparison to available transcriptomic sequences (25). To identify or confirm RSS sequences, an RSS information content model (RIC) was used (26; https://www.itb.cnr.it/rss/index.html). NCBI’s BLASTp or tBLASTn algorithms were used to confirm V sequences and assess their similarity to TCR homologs from various species retrieved from GenBank (27). V gene nucleotide segments were then aligned with ClustalW (28). Gene segments were annotated following the international ImMunoGeneTics information system nomenclature (29). Gene segments were named according to their location from 5’ to 3’ end on the locus. V gene families were defined by sharing ≥80% nucleotide sequence identity based on ClustalW alignments (28).

### Phylogenetic analysis

MEGAX was used to convert nucleotide sequences for both variable (V) genes and constant region genes (C) into amino acid residues which were then aligned with MUSCLE (30, 31). The aligned sequences were then used to construct phylogenetic trees using the neighbor-joining method (32). The trees were then visualized using iTOL (33).

Variable and constant gene sequences with accession numbers used in all phylogenetic analyses are found in **Supplementary Tables 2–5**. *S. punctatus* TRBV are found on scaffold QEPC01009940.1 (https://www.ncbi.nlm.nih.gov/). The Chinese alligator (*Alligator sinensis) TRBV* sequences were provided by Wang et al. (34). Opossum (*Monodelphis domestica*) *TRB* are also found in Parra et al. (35). *Xenopus tropicalis* and *Ambystoma mexicanum TRBV* sequences were provided by Jesus Martinez.

### Percent nucleotide identity matrix

Germline nucleotide sequences were collected from both *T. rugosa and S. punctatus* (see above). Sequences were aligned via ClustalW (28). Gene segments were annotated following IMGT nomenclature. Families were defined by having ≥80% nucleotide identity in the ClustalW alignment (28). Analysis and visualization of the percent identity matrix generated by ClustalW was conducted using the R packages ggplot2 and reshape2 (28; RStudio 2024.4.2.764; 36–39).

### Constant region analysis

TCR constant region sequences from *Gallus gallus TRAC* (MN646854.1), *Gallus gallus TRBC* (BAC67174), *S. punctatus TRBC* (GGNQ01096868.1), *S. punctatus TRGC* (GGNQ01074423.1), *S. punctatus TRDC* (GGNQ01087842.1), and *T. rugosa TRAC* (UYS90863.1), *T. rugosa TRBC* (UYS90848.1), and *T. rugosa TREC* were aligned via ClustalW (28). Sequences were then analyzed for transmembrane regions using DeepTMHMM-2.0 (23).

## Results

**Figure 1:**
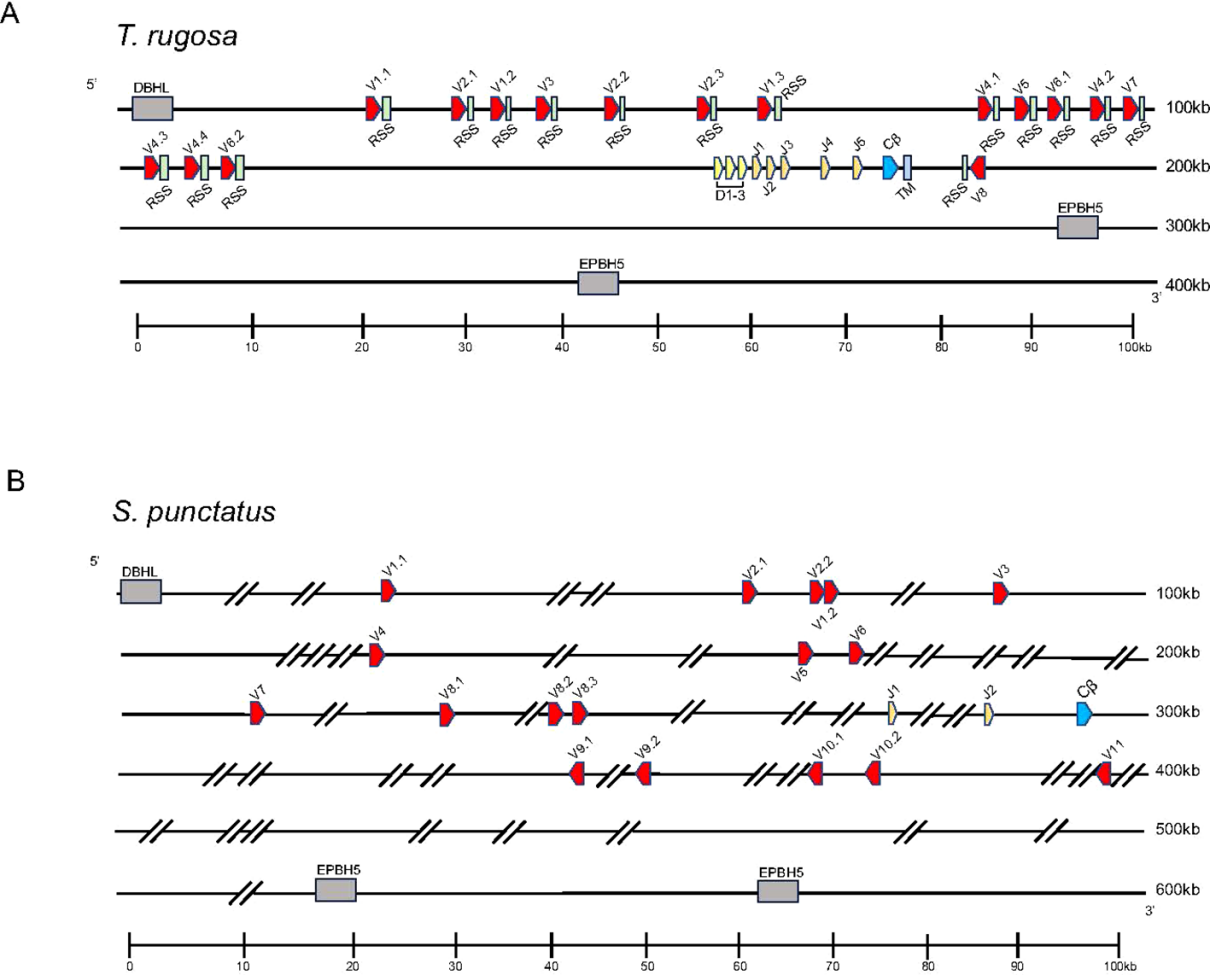
Map of the *TRB* loci. *TRBV* designated V (red), *TRBD* designated D (yellow), *TRBJ* designated J (orange), *TRBC* designated Cβ (blue), and the TRB transmembrane region designated TM (light blue) gene segments are numbered by their corresponding location in order across the locus. 23bp spacers are shown in light green near V segments. Other RSS are not shown for space. The flanking genes on the 5’ end of the locus was DBH-like monooxygenase protein 2 (*DBHL*). The 3’ flanking gene is ephrin type-b receptor 5-like (*EPHB5-like*). V segments are designated with a family member number followed by a period and a designated number according to their gene family. Transcriptional orientation is indicated by the direction of the arrow on each gene segment or exon. Arrows are not proportionate to the actual gene sizes. Gray boxes indicate flanking genes. Gaps in the genome are indicated by hatch marks. **(A)** Shows the skink TCR β locus in pseudohaplotype 2. The locus is ~373kb in length. Shown in the TRB locus on haplotype 2. **(B)** Shows the tuatara TCR β locus. The locus ~718kb in length.

**Figure 2:**
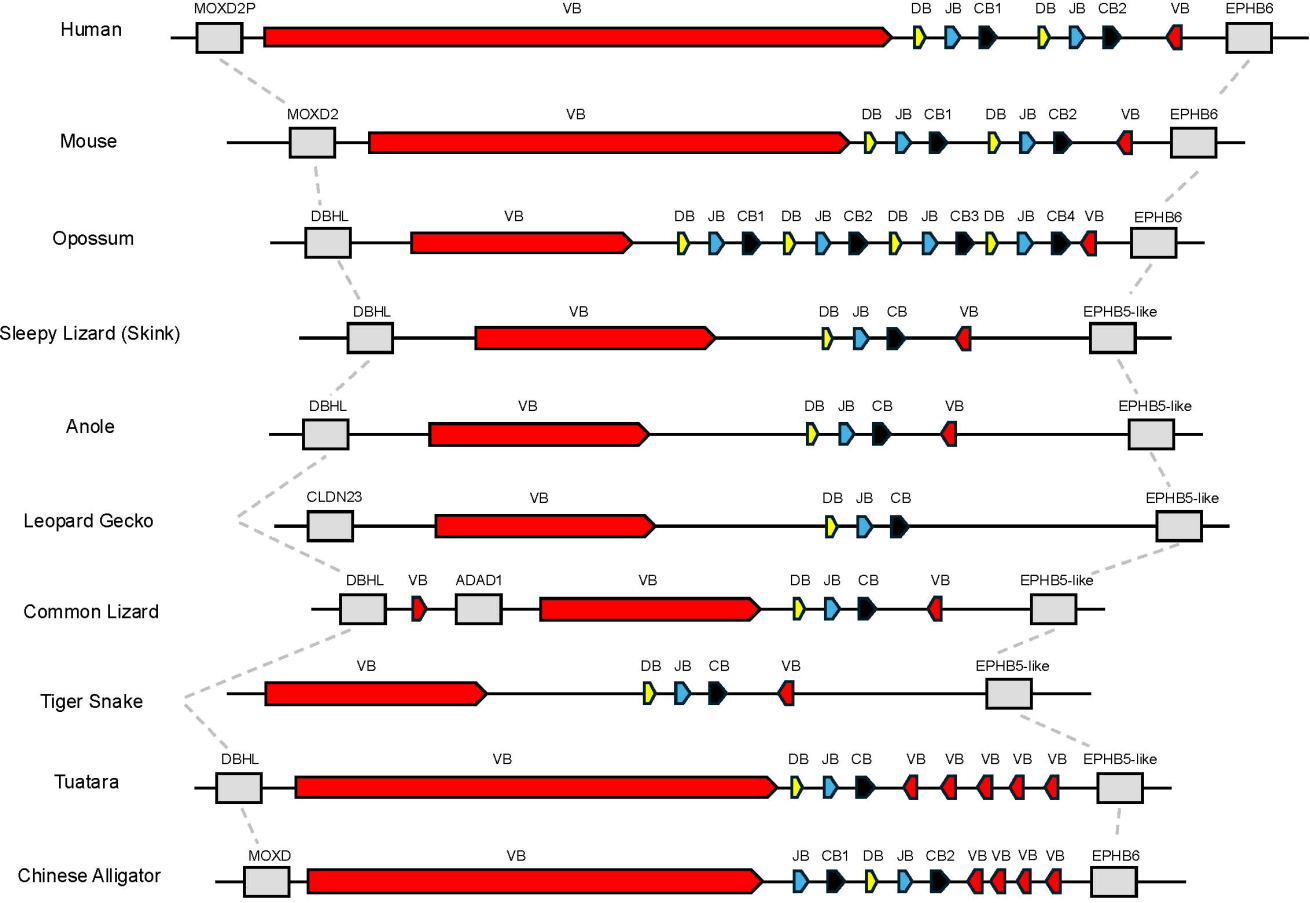
Comparison of the region containing *TRB*. Dashed lines connect genes with conserved synteny in this genomic region between species shown. Flanking genes are shown in grey. Monooxygenase, DBH-like 2 (*MOXD2P/MOXD2/DBHL*), ephrin type B receptor 6 or ephrin type b receptor 5-like (*EPHB6* or *EPHB5-like*), claudin 23 (*CLDN23*), and adenosine deaminase containing protein 1 (*ADAD1*) are shown. Regions containing *TRBV* genes are labeled as VB shown in red, *TRBD* are labeled as DB shown in yellow, *TRBJ* are labeled as JB shown in blue, and *TRBC* are labeled as CB shown in black.

Initially, we set out to characterize the *TRB* loci in the skink *T. rugosa* using the tuatara *S. punctatus* for comparative purposes. The *T. rugosa TRB* locus is located on chromosome 2 and is approximately 373kb in length (**Figure 1A**). The *S. punctatus TRB* locus is at least 718kb in length (**Figure 1B**). There is conserved synteny surrounding the *TRB* loci, which are flanked by *DBHL* (DBH-like monooxygenase protein 2) at the 5’ end and *EPHB5-like* (ephrin type-b receptor 5-like) at the 3’ end in both species (**Figures 1**, **2**). This conserved synteny is maintained in several amniote species (**Figure 2**) (34, 35, 40–43).

Both available *T. rugosa TRB* pseudohaplotypes were annotated and found to contain 15 and 16 *TRBV* gene segments in pseudohaplotypes 1 and 2, respectively. These gene segments could be classified into eight families based on nucleotide identity (**Supplementary Figure 1A**). All families were found in both pseudohaplotypes. Noteworthy was a single *TRBV* gene segment in an inverted reading frame relative to the rest of the locus on the 3’ side of *TRBC* (**Figure 1A**). As will be discussed later, inverted *TRBV* at the extreme 3’ end of the *TRB* locus is a feature shared with many other amniote species. Both *T. rugosa* pseudohaplotypes contained three *TRBD*, six *TRBJ*, and a single *TRBC* gene (**Figure 1A**). The *T. rugosa TRBV* sequences were flanked by a 23 base pair (bp) spacer and canonical CACAGTG heptamer (**Figure 1A**; 25, 44). The *TRBD* gene segments were flanked by a 12 bp spacer on the V proximal side and a 23 bp spacer on the C proximal side. Similarly, the *TRBJ* segments were flanked by a 12 bp spacer (not shown). In *T. rugosa* 100% of the RSS flanking the TRB V, D, and J segments were canonical (not shown). In other squamate species, the RSS appeared uniformly non-canonical e.g. CACAGCA (not shown). However, non-canonical RSS have been routinely shown to be functional (44). Across a wide variety of vertebrates, there is nucleotide conservation of *TRBD* genes (45). The most V proximal *T. rugosa* D segment, *TRBD1*, contains this conserved sequence (GGGACAGGGGGC) and is identical to *TRBD* sequences found in the *A. carolinensis*, the common lizard (*Zootoca vivipara*), and the mainland tiger snake (*Notechis scutatus*) (**Supplementary Table 6**).

*Sphenodon punctatus* has 17 *TRBV* gene segments which are classified into 11 gene families based on nucleotide identity (**Figure 1B**; **Supplementary Figure 1B**). Furthermore, five *TRBV* genes were inverted and found on the 3’ side of the single *TRBC* gene (**Figure 1B**). Two *TRBJ* gene segments were identified in *S. punctatus*, but no *TRBD* gene segments could be identified in the current genome, most likely due to gaps in the genome assembly (**Figure 1B**).

**Figure 3:**
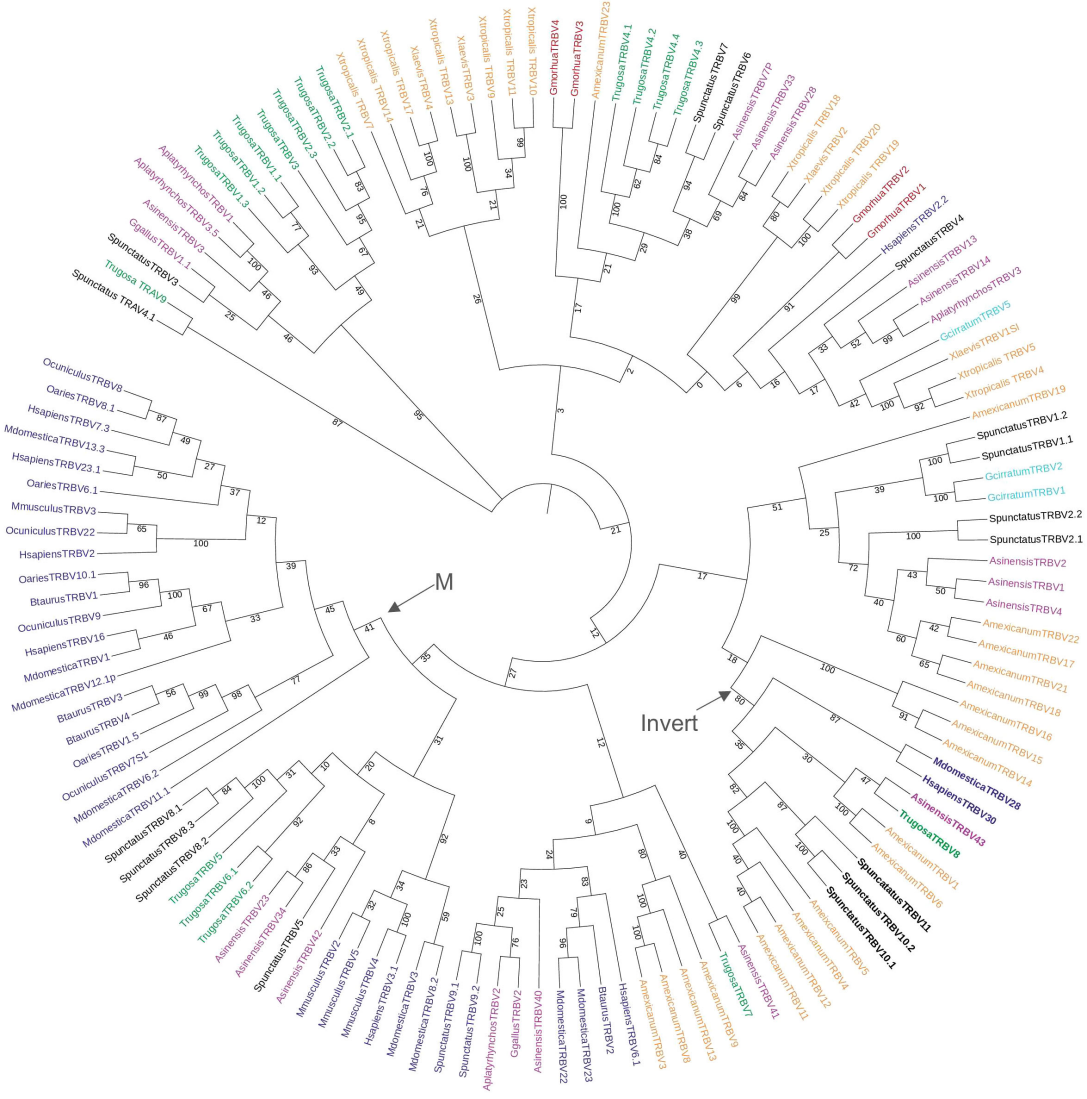
Neighbor-joining tree of vertebrate *TRBV* genes based on an amino acid alignment of *TRBV* sequences. Numbers on branches indicate bootstrap values on 1,000 replicates. Tree containing *TRBV* from 6 mammals, 1 squamate reptile, 1 Rhynchocephalian, 3 archelosaurs, 2 amphibians, 1 teleost fish, and 1 cartilaginous fish. The clade containing the inverted *TRBV*s is bolded and labeled with an arrow and “Invert”. Mammalian specific clade marked by arrow and “M.” *TRBV* families of squamates intersperse amongst the tree and 3’-inverted *TRBVs* consistently cluster together. Mammals included were short-tailed opossum (*M. domestica*), human (*H. sapiens*), mouse (*M. musculus*), cow (*B. taurus*), sheep (*O. aries*), and rabbit (*O. cuniculus*); the squamate is skink (*T. rugosa*); the Rhynchocephalian is tuatara (*S. punctatus*); the archelosaurs are Chinese alligator (*A. sinensis*), chicken (*G. gallus*), and duck (*A. platyrhynchos*); the amphibians are axolotl (*A. mexicanum*), African clawed frog (*X. laevis*), and Western clawed frog (*X. tropicalis*); the teleost is cod (*G. morhua*), and the cartilaginous fish is nurse shark (*G. cirratum*). Accession numbers of sequences used in the tree are found in **Supplementary Table 2**.

We compared the *T. rugosa* and *S. punctatus TRBV* sequences to *TRBV* of other vertebrate species in a phylogenetic analysis (**Figure 3**). *TRBVs* of both *T. rugosa* and *S. punctatus* were interspersed amongst the V genes of other vertebrates consistent with *TRBV* germline diversity being evolutionarily ancient (**Figure 3**). The exception is one clade that includes only mammalian *TRBV* (**Figure 3**). The 3’-inverted *TRBV* formed their own clade in the phylogenetic analysis despite low bootstrap values in multiple iterations of the tree including minimum evolution and maximum likelihood (**Figure 3**; **Supplementary Figure 3A**). This is consistent with a common ancestral inversion. We note that several amphibian *TRBV* from the axolotl *Ambystoma mexicanum* that are not inverted, also fall into this clade, whereas non-inverted *TRBV* from *Xenopus tropicalis* did not (**Supplementary Figure 3**).

There were 38 *TRB* transcripts identified from the two *T. rugosa* spleen transcriptomes. Of those 38 sequences, 20 (52.6%) were complete enough at the 5’ end to show evidence of V(D)J recombination. Of those 20 transcripts, 16 (80%) were productively rearranged (**Supplementary Figure 2A**). The remaining transcripts contained out of frame V(D)J rearrangements that would not encode a functional TRB V domain.

**Figure 4:**
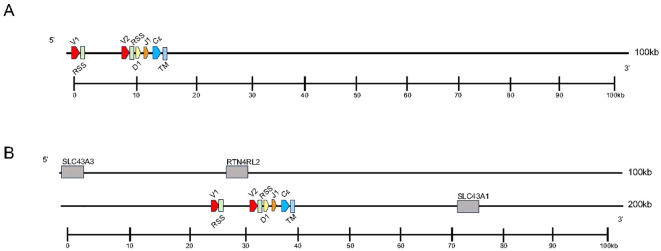
Map of the skink *TRE* locus. *TREV* (red), *TREJ* (orange), *TRED* (yellow), *TREC* (blue), and the *TRE* transmembrane region (light blue) gene segments are numbered by their corresponding location in order across the locus (nomenclature as seen in **Figure 1**). 23bp spacers are shown in light green near V segments. Other RSS are not shown for space. *TREV* segments are designated with a family member number. Transcriptional orientation is indicated by the direction of the arrow on each gene segment or exon. Arrows are not proportionate to the actual gene sizes. Gray boxes indicate flanking genes. **(A)** The skink *TRE* locus is ~16kb in length from V1 to TM region. **(B)** The flanking genes on the 5’ end of the locus are equilibrative nucleobase transporter 1 (*SLC43A3*) and reticulon-4 receptor-like 2 (*RTN4RL2*). The 3’ flanking gene is large neutral amino acids transporter small subunit (*SLC43A1*). The syntenic block containing *TRE* and the flanking genes is ~176kb.

Analysis of the *T. rugosa* genome uncovered the presence of a third putative *TCR* locus similar to that recently described by Gambón-Deza, who designated it as TCR epsilon (TCRϵ or *TRE*) (18). The *T. rugosa TRE* locus is on chromosome 1 and is approximately 16kb in length from the most 5’ V to the 3’ C (**Figure 4A**). It contains 2 *TREV*, 1 *TRED*, 1 *TREJ* gene segments, and a single *TREC* in both pseudohaplotypes. As with the *TRBV*, the *T. rugosa TREV* sequences were both flanked by a 23 bp spacer and canonical heptamer (**Figure 4**; 25). The *T. rugosa TRED* gene segment was flanked by a 12 bp spacer on the V proximal side and a 23 bp spacer on the J proximal side. The *T. rugosa TREJ* segment was flanked by a 12 bp spacer (not shown). This pattern of spacers in the *TRE* locus is the same in several squamate species examined save for *A. carolinensis* (not shown)*. A. carolinensis* had Vs flanked by both 12 bp spacers and 23 bp spacers and Js similarly flanked by both 12 and 23 bp spacers, demonstrating inversions that took place in the *A. carolinensis TRE* locus (not shown). We were unable to identify *A. carolinensis TRED* gene segments and therefore don’t know their RSS types (not shown, **Supplementary Table 6**). *TRE* was found in the genomes of *Gekkonidae*, *Phrynosomatidae*, *Varanidae*, *Elapidae*, *Scincidae*, *Dactyloidae*, *Lacertidae*, and *Amphisbaenidae* and was likely present in the last common ancestor of Squamates (**Supplementary Table 6**). In comparison to the genomes of other squamates, the *T. rugosa TRE* locus has among the lowest number of *TREV* gene segments (**Table 1**; **Supplementary Table 6**).

**Table 1:** *TRB* and *TRE* V comparison between multiple species.

Common Name	Species	TRBV	TREV	Total Vs	Reference
Tuatara	*Sphenodon punctatus*	17	0	17	Current Study; (16)
Sleepy Lizard (Skink)	*Tiliqua rugosa* *(Scincidae)*	15/16^a^	2	17-18	Current Study; (16)
Green Anole	*Anolis carolinensis* *(Dactyloidae)*	7	4	11	Current Study; (46; 18)
Leopard Gecko	*Eublepharis macularius* *(Gekkonidae)*	9	1	10	Current Study; (46; 18)
Common Lizard	*Zootoca vivipara* *(Lacertidae)*	8	5	13	Current Study
Komodo Dragon	*Varanus komodoensis* *(Varanidae)*	2	1	3	Current Study: (18)
Water Monitor	*Varanus salvator* *(Varanidae)*	2	ND	2	Current Study
Mainland Tiger Snake	*Notechis scutatus* *(Elapidae)*	4	2	6	Current Study; (18)
Fence Lizard	*Sceloporus undulatus* *(Phrynosomatidae)*	10	1	11	Current Study
Florida Worm Lizard	*Rhineura floridana* *(Amphisbaenidae)*	8	4	12	Current Study

a Depending on haplotype.

There were 40 TRE sequences identified in two *T. rugosa* spleen transcriptomes. Twenty two of the 40 sequences (55%) were complete enough at the 5’ end to have evidence of being transcribed from a *TRE* locus that had undergone V(D)J recombination. Only three of the 22 (13.6%) were productively rearranged (**Supplementary Figure 2B**). Both *TREV* gene segments were found to be used in rearrangements (**Supplementary Figure 2B**). The majority of the transcripts contained out of frame V(D)J rearrangements that would not encode a functional TRE V domain.

**Figure 5:**
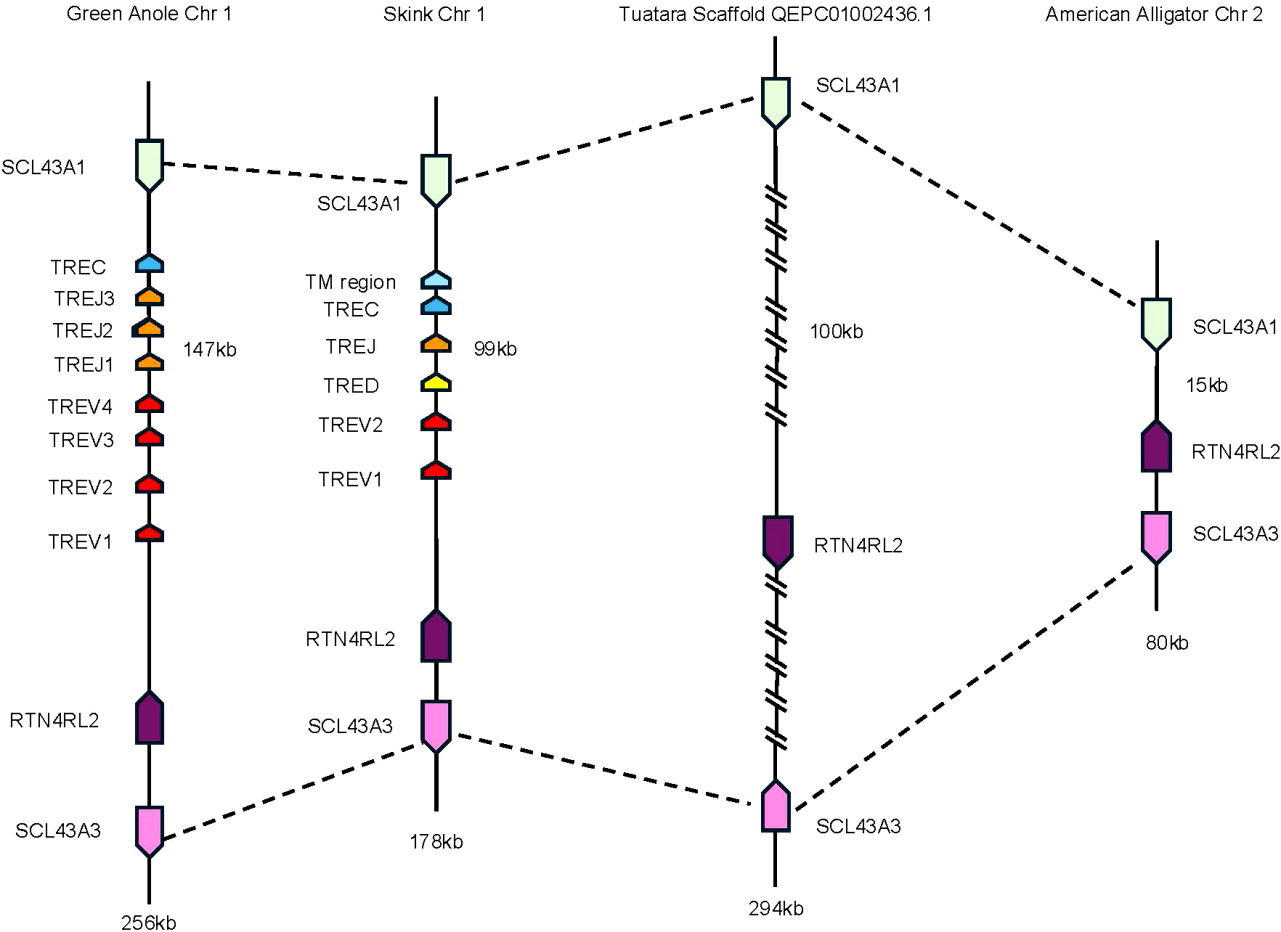
Comparison of the region containing *TRE*. Dashed lines connect genes with conserved synteny between the skink and tuatara. Genes flanking the *TRE* locus in the skink are equilibrative nucleobase transporter 1 (SLC43A3) (pink) and reticulon-4 receptor-like 2 (RTN4RL2) (dark purple) on the 5’ end and large neutral amino acids transporter small subunit (SLC43A1) (light green) on the 3’ end. The skink *TRE* locus is located between these genes (nomenclature as in **Figures 1**, **3**). These same flanking genes were also identified in the tuatara, anole, and American alligator. Distance between RTN4RL2 and SCL43A1 shown next to each locus. The absence in the tuatara is either due to the absence of the locus or due to gaps in the scaffold. Gaps are indicated by hatch marks. Lengths of the complete loci are shown beneath each organism.

To investigate the evolutionary origins of *TRE*, we searched for areas of synteny in the genomes of non-squamate reptiles, which lack *TRE*, compared with squamate *TRE*. In *T. rugosa*, *TRE* is flanked by *RTN4RL2* (reticulon-4 receptor-like 2) and *SLC43A3* (equilibrative nucleobase transporter 1) on the 5’ side and *SLC43A1* (large neutral amino acids transporter small subunit 3) on the 3’ side (**Figure 4B**). This syntenic block was conserved in all reptiles examined (**Figure 5**). In *T. rugosa*, the flanking genes are 99 kb apart (**Figure 5**). In contrast, in the American alligator (*Alligator mississippiensis*), the distance between these genes is only 15kb (**Figure 5**). *TRE* could not be identified in the current *S. punctatus* genome, although absence due to gaps in the current assembly could not be ruled out (**Figure 5**). However, we had no difficulty identifying the *S. punctatus TRA/D*, *TRB*, and *TRG* loci (**Figure 1B**; 16). Moreover, we were unable to find *TRE* transcripts in an available *S. punctatus* blood transcriptome dataset, even though there was no difficulty identifying *TRA*, *TRD*, *TRB*, and *TRG* transcripts in this same dataset (**Supplementary Table 1**; 16).

**Figure 6:**
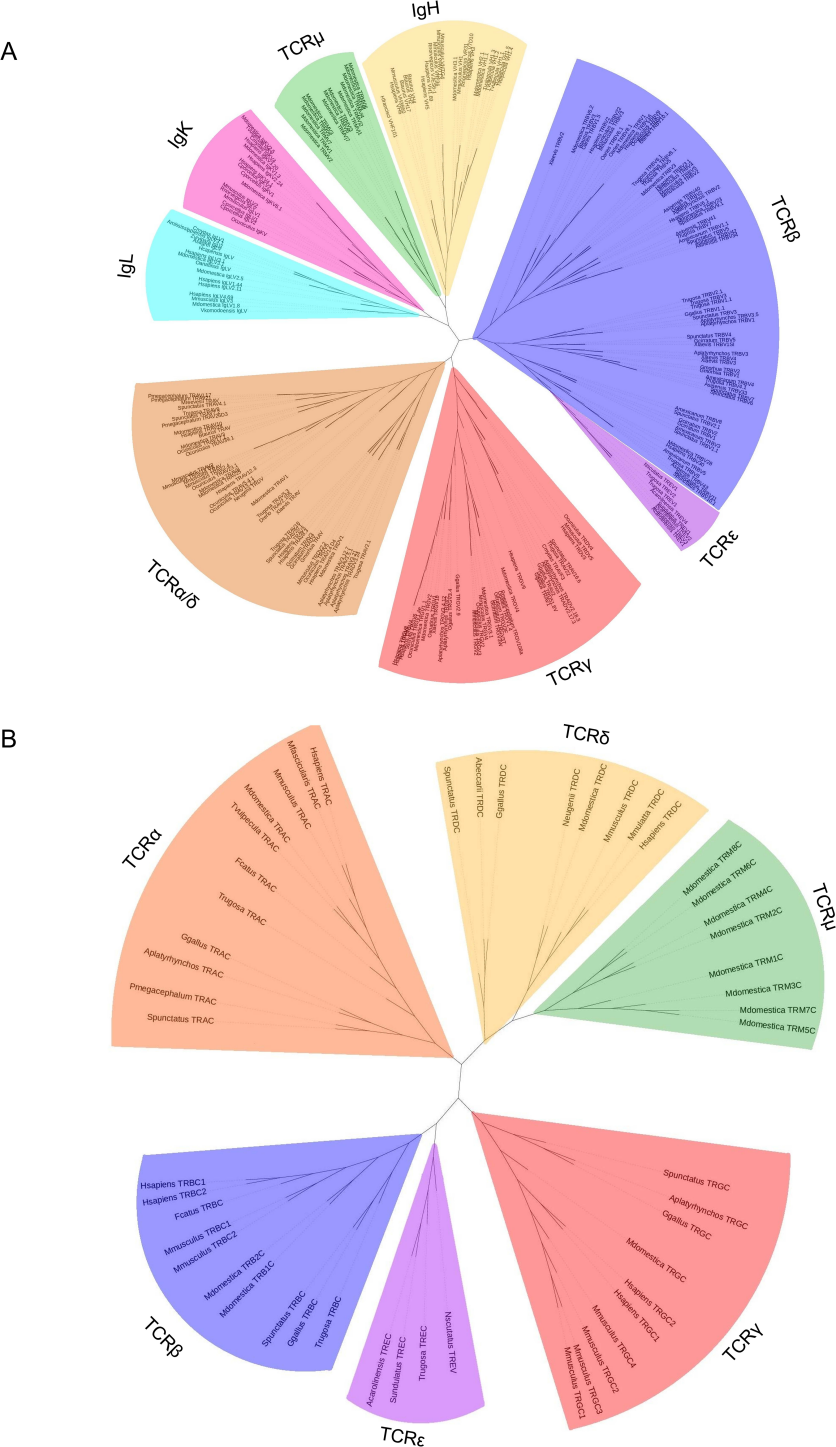
Neighbor-joining trees based on amino acid alignments of vertebrate variable (V) genes and constant regions (C). **(A)** V genes from both TCRs and Igs from several species. Vs from IgL are shown in light blue, IgK are shown in pink, IgH are shown in gold, TCRμ are shown in green, TCRβ are shown in light purple, TCRϵ are shown in dark purple, TCRγ are shown in red, and TCRα/δ are shown in red. **(B)** TCR constant regions from multiple species. Cs from TCRμ are shown in green, TCRγ are shown in red, TCRϵ are shown in dark purple, TCRβ are shown in light purple, TCRα are shown in orange, and TCRδ are shown in gold. Mammals included are humans (*H. sapiens*), crab eating macaque (*M. fascicularis*), sheep (*O. aries*), cow (*B. taurus*), pig (*S. scrofa*), rabbit (*O. cuniculus*), rat (*R. norvegicus*), cat (*F. catus*), short-tailed opossum (*M. domestica*), Tammar wallaby (*N. eugenii*), brushtail possum (*T. vulpecula*), and platypus (*O. anatinus*); squamates included are skink (*T. rugosa*), anole (*A. carolinensis*), mainland tiger snake (*N. scutatus*), and fence lizard (*S. undulatus*); the Rhynchocephalian is tuatara (*S. punctatus*); the archelosaurs are Chinese alligator (*A. sinensis*), Western bronze ground-dove (*A. beccarii*), chicken (*G. gallus*), duck (*A. platyrhynchos*), big headed turtle (*P. megacephalum*), Reeve’s turtle (*M. reevesii*), and green sea turtle (*C. mydas*), the amphibian is African clawed from (*X. laevis*), the teleost fish are cod (*G. morhua*), and zebrafish (*D. rerio*); and the cartilaginous fish are nurse shark (*G. cirratum*), and horned shark (*H. francisci*). Accession numbers of sequences used in 6A and 6B are found in **Supplementary Tables 3** and **4** respectively.

*TREV* genes were compared to other V genes found in immune receptors. There are five known TCR loci in amniotes, *TRA, TRB, TRG, TRD*, and *TRM*, and V genes from all five were included in the analysis (14, 35, 47; **Figure 6A**). Also included were V genes from the immunoglobulin heavy chain locus and both amniote light chain loci, kappa and lambda (**Figure 6A**). *TREV* consistently clustered with *TRBV* genes in multiple iterations of the tree including maximum likelihood and minimum evolution trees (**Figure 6A**; **Supplementary Figure 3**). Specifically, *TREVs* are the sister lineage to the 3’-inverted *TRBV* gene segments (**Figure 6A**; **Supplementary Figure 3**). We also compared the gene encoding the constant (C) domain of TRE to the C regions of the other five TCR, and it was most related to the C region genes encoding the TCRβ constant region (**Figure 6B**).

**Figure 7:**
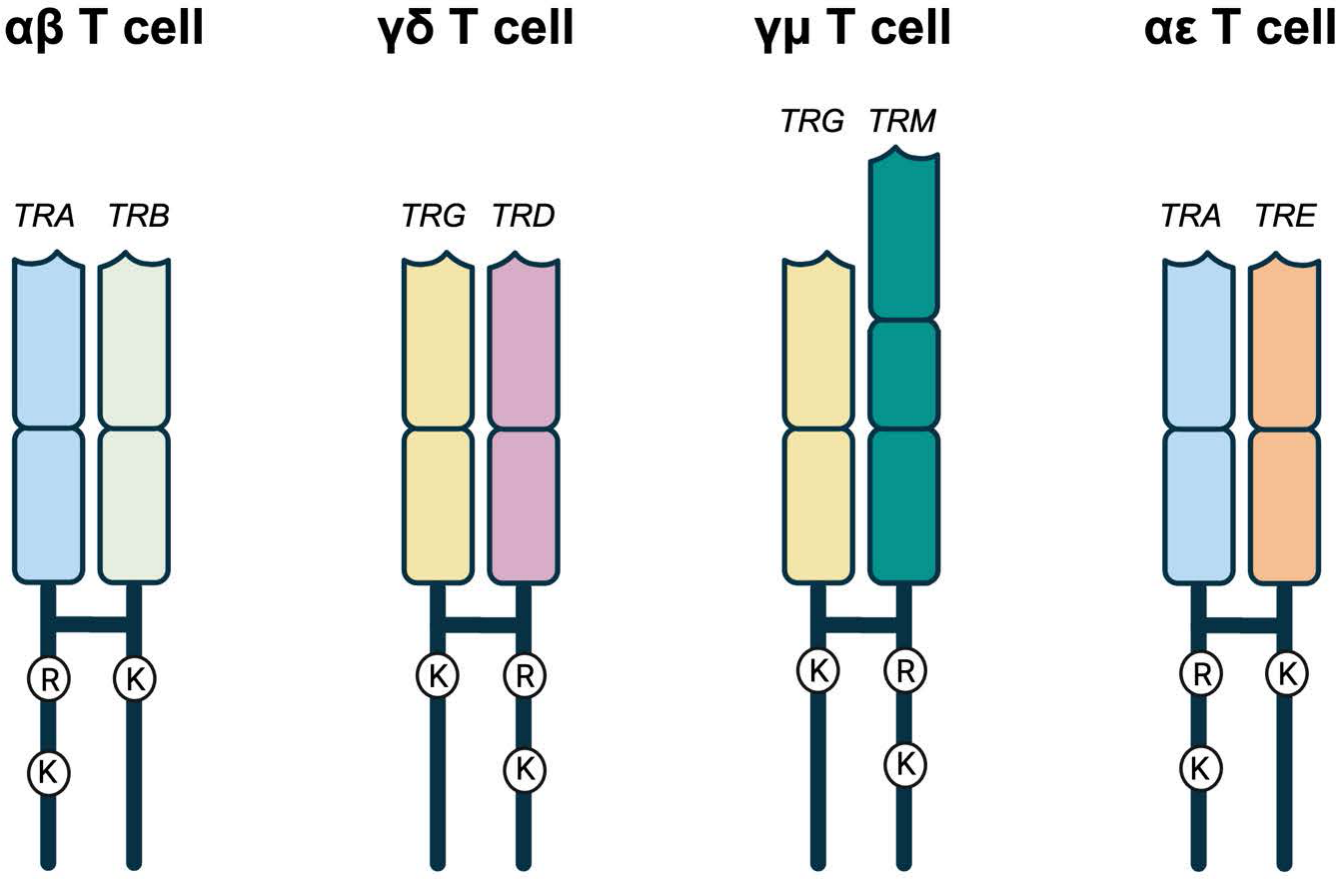
Representative TCRs with the amino acids in their transmembrane regions. αβ, γδ, and γμ represent the known amniote T cell receptors. In all three, there is an asymmetry of amino acids found in the transmembrane regions. One of the TCRs has an arginine (R) and a lysine (K) while the other has a single lysine (K). *TRA*, *TRD*, and *TRM* all have the R and K while *TRB* and *TRG* have the single K. In the potential squamate TCR, *TRA* still has the R and K while *TRE* has the single K that would allow it to potentially pair with *TRA* allowing for the possibility of αϵ T cells. Created in BioRender. Miller, R (2025). https://BioRender.com/v19g154.

Given *TRE* appears most related to *TRB*, we predicted the TCRϵ chain would likely pair with TCRα. For proper TCR heterodimer formation and interaction with the CD3 signaling complex, each TCR chain contains conserved arginine (Arg) and lysine (Lys) residues in the transmembrane region (47, 48). These conserved residues have an asymmetric pattern in the heterodimer, where one chain contains both Arg and Lys, while the other only Lys (**Figure 7**; **Supplementary Figure 4**) (47, 48). In a conventional αβTCR pair, the TCRα has Arg/Lys and the TCRβ has Lys only (**Figure 7**; **Supplementary Figure 4**). The same is true of squamate αβTCR (**Figure 7**; **Supplementary Figure 4**). The translated TCRϵ sequence has a conserved Lys at position 768, which is consistent with its ability to pair with TCRα and create a the TCR-CD3 complex (**Figure 7**; **Supplementary Figure 4**; 18).

## Discussion

Squamate reptiles are amongst the most successful vertebrate lineages. More than 10,000 species occupy a broad range of ecosystems, from sea snakes to desert horned lizards. Despite their broad distribution and diversity, the squamates, and reptiles in general, remain amongst the least studied vertebrate lineages with respect to their immune systems, a shortcoming noted two decades ago (49). Indeed, most Sauropsid immunology has focused on a small number of species, mostly Archelosaurs, and has largely excluded the Lepidosaurs (9–12). What is known of reptile immune responses has primarily centered on innate immune responses with the conclusion that they may depend less on the adaptive response (11). Thankfully, the tools of genomics have increased the accessibility of many species to investigation, substantially enhancing the field of comparative biology, including comparative immunology.

The Australian skink species, *T. rugosa*, has several characteristics useful for a model squamate. They are abundant, widely distributed, and there is a 40 plus-year record of pathogen studies (50–54). *Tiliqua rugosa* is a host to multiple tick species that have been found to be vectors for blood pathogens such as rickettsia and apicomplexan protozoans (53–56). In the past, these tick species occupied distinct ecological zones (56). The tick boundary is known to shift between drier and wetter years, demonstrating how climate change might influence pathogen distribution (52, 56, 57).

We previously reported the lack of γδ T cells in squamates was due to deletions of the *TRG* and *TRD* loci needed to encode the TCRγ and TCRδ chains, respectively (16). Here, we investigate how the absence of the TCRγ and TCRδ chains may have influenced the remaining TCR genes. Our previous work showed little increase in the complexity of the *TRA* locus at the genomic level in the *T. rugosa* (16). Indeed, there is a relative decrease in complexity in the *T. rugosa TRA* locus, relative to *S. punctatus* which retains the TCRγ and TCRδ chains. Overall, there is comparatively low complexity in the available *TRBV* genes needed to assemble the exon encoding the TCRβ variable domain. Low numbers of *TRBV* genes appears to be the norm for Lepidosaurs (21, 46, 58). It is unlikely that an increase in the clonal diversity of αβ T cells, therefore, compensates for the loss of γδ T cells in squamates.

Surprising was the discovery that squamates have an additional locus that contains V, D and J segments like the genes encoding the conventional TCR and Ig. The *T. rugosa* locus is clearly homologous to a locus described recently by Gambón-Deza, who designated it as T cell receptor epsilon (*TRE;* 18). Analyses of the *T. rugosa TRE* gene segments are consistent with it being from a partial duplication of the *TRB* locus.

**Figure 8:**
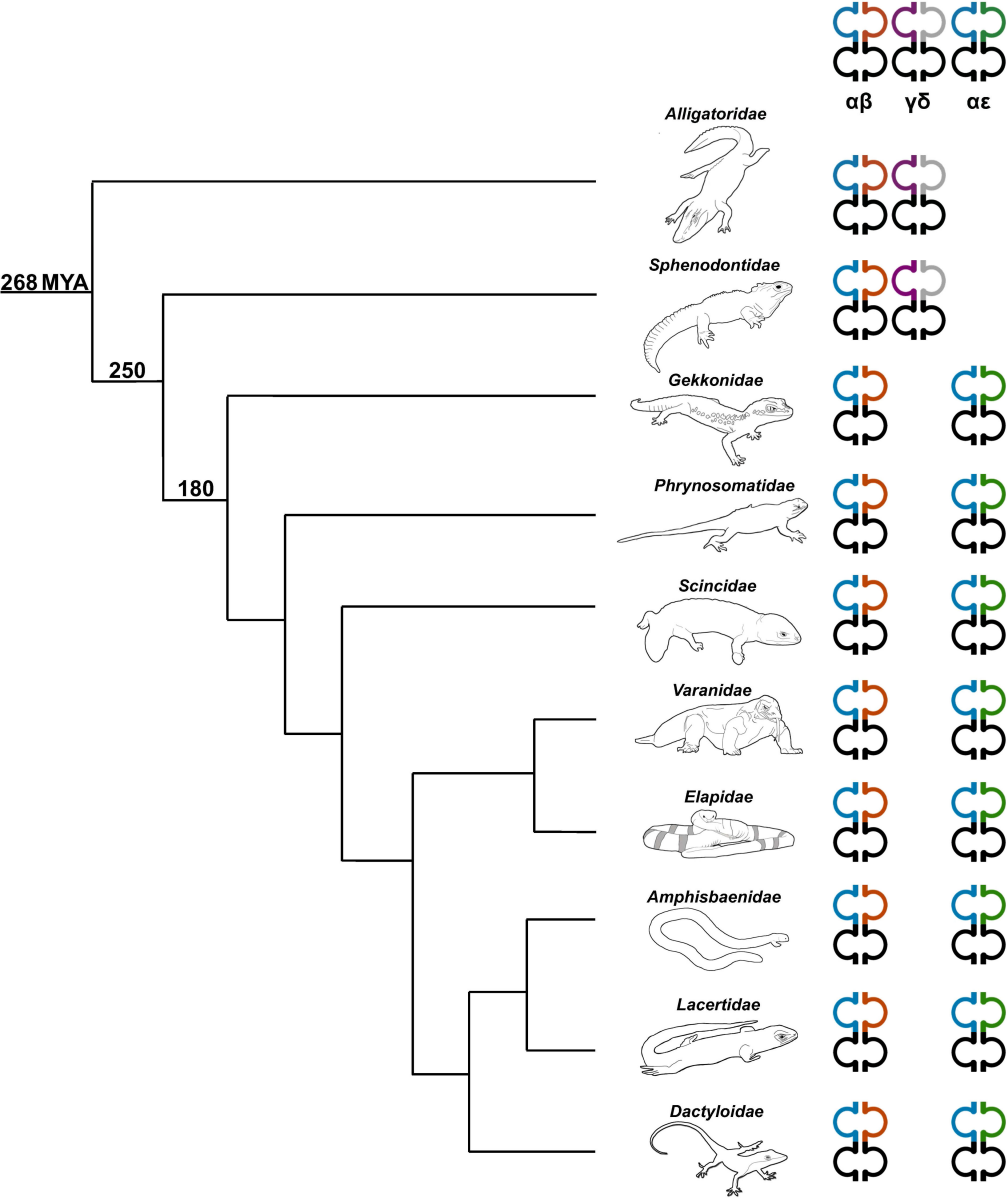
Phylogenetic relationship illustrating the diversity of TCR content in representative sauropsids. Representatives from several families were used including the Florida worm lizard (*Amphisbaenidae*), common lizard (*Lacertidae*), green anole (*Dactyloidae*), Komodo dragon (shown) and water monitor (not shown) (*Varanidae*), mainland tiger snake (*Elapidae*), skink (*Scincidae*), fence lizard (*Phrynosomatidae*), leopard gecko (*Gekkonidae*), tuatara (*Sphenodontidae*), and American alligator (*Alligatoridae*) (59). The number on each clade indicates approximate predicted divergence times in millions of years (MYA) (2, 5, 60). Heterodimer pairs are indicated at the top of each TCR chain type. *TRA* is shown in blue, *TRB* is shown in orange, *TRG* is shown in purple, *TRD* is shown in grey, and *TRE* is shown in green.

**Figure 9:**
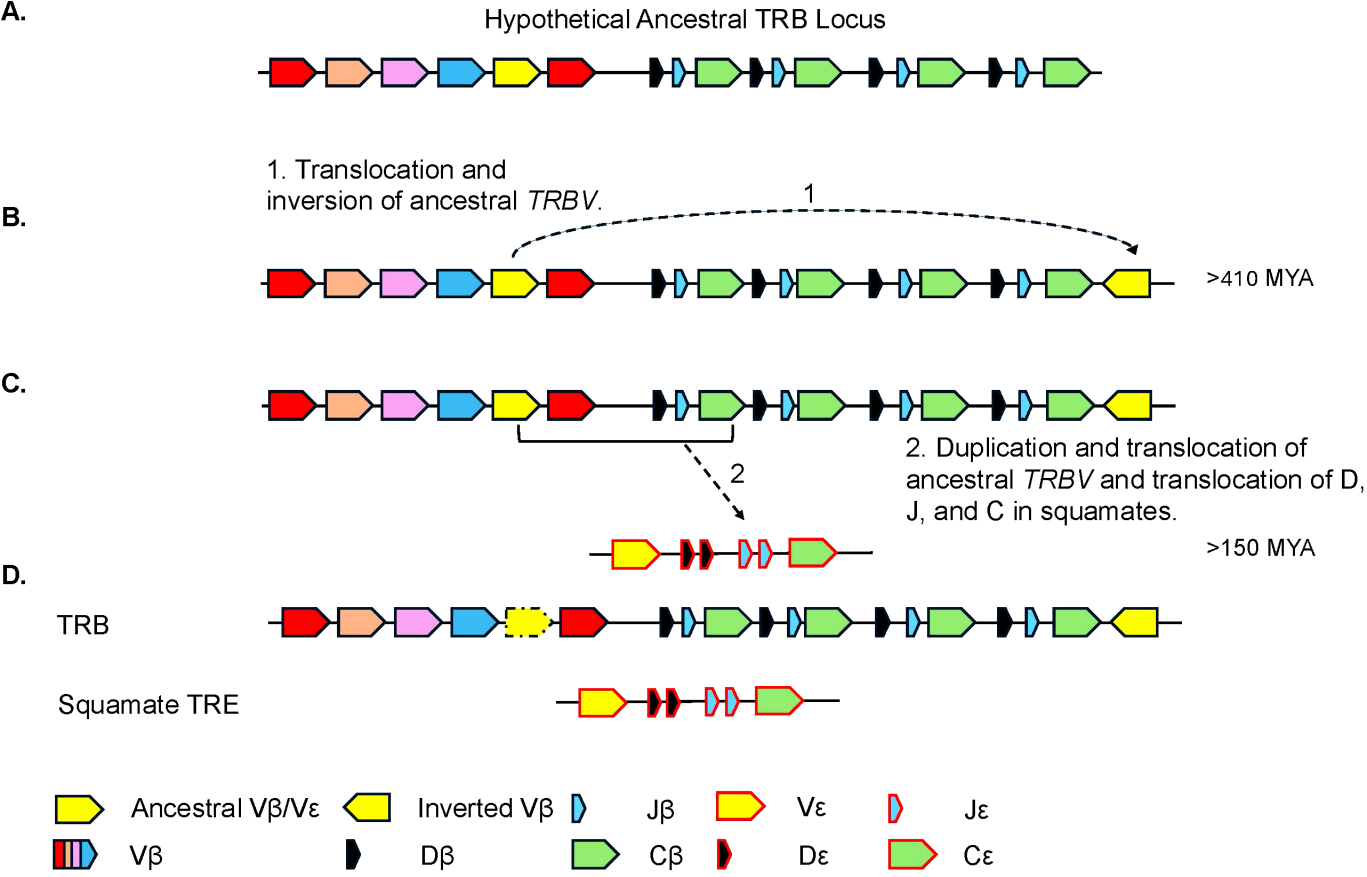
Proposed model for the evolution of the *TRB* and *TRE* loci. **(A)** Proposed model of the ancestral TRB locus. Highlighted in yellow is the *TRBV* gene segment(s) that is/are ancestral to the extant inverted *TRBV* and *TREV* gene segments. Other *TRBV* families are shown in additional colors. **(B)** Model hypothesizing (arrow 1) the duplication and inversion of the *TRBV* gene segment(s) within the *TRB* locus, currently found in several species. **(C)** The duplication and translocation of the TRB V-D-J-C (arrow 2) region to create the TRE locus. **(D)** Generic common *TRB* locus including the locus found in squamates and the squamate specific *TRE* locus. Presence of *TRBV* gene that gave rise to the inversion is species dependent and shown with dashed arrow.

*TRE* was only found in the genomes of squamates, which lack γδ T cells, and not in non-squamate reptiles like *S. punctatus*, and *A. mississippiensis* (**Figure 8**) (16). This is consistent with the duplication giving rise to *TRE* occurring after the split between Rhynchocephalia and Squamata 250-280 MYA, and prior to the divergence of squamates more than 150 MYA (2, 5, 61). Analysis of the *TREV* genes revealed their relationship to a clade of *TRBV* that are in an inverted orientation and 3’ position in the *TRB* locus of most amniotes (34, 35, 40, 42, 43, 62). This inversion is also found in salmonids and some amphibians, consistent with it occurring earlier in vertebrate evolution (63; Jesus Martinez personal communication). From these observations emerges a model for the evolution of the *TRB* locus in amniotes and the origin of the *TRE* locus in squamates (**Figure 9**). Beginning with an ancestral *TRB* locus (**Figure 9A**) a family of TRBV translocated to an inverted location 3’ of the constant region genes (**Figure 9B**). Within the squamates, there was a translocation of a cluster of TRBV-D-J-C genes likely to another giving rise to *TRE* (**Figure 9C**). These duplications and translocations have resulted in the current conventional *TRB* locus in all amniotes and *TRE* in squamates (**Figure 9D**).

Inverted V gene segment(s) are common to amniote *TRB* loci, are recombined in the αβ T cell repertoires, and detectable in transcriptomes. (34, 35, 40, 42, 43, 62–66; **Supplementary Figure 2A**). Indeed, inversions of genomic regions at the Ig and TCR loci are not uncommon throughout evolution (64, 65). Therefore, it is not clear if there is a fitness advantage to having these inverted V genes. The evidence that the *TREV* are most related to the inverted *TRBV* may simply reflect the plasticity of the locus that gave rise to the inversions in the first place locus that gave rise to the inversions in the first place.

In conventional T cells, the pairing of TCRα with TCRβ and TCRγ with TCRδ appears strictly enforced and remarkably conserved (14, 67, 68). However, there is precedence for T cell receptor gene duplications giving rise to novel TCR forms. To date, these novel TCR forms have involved specifically duplications of the *TRD* locus. In some birds, the *TRD* locus has been duplicated with the second locus using antibody heavy chain V gene segments in place of conventional *TRVD* (69, 70). Although it has not been physically demonstrated, it is likely the chains encoded by this second *TRD* locus also pair with the TCRγ chain. In mammals, duplications of the *TRD* locus gave rise to the genes encoding the T cell receptor μ chain (47, 71). The TCRμ chain has an unusual structure by having three extracellular immunoglobulin domains, however TCRμ has been shown to physically pair with TCRγ creating the γμTCR (72). γμ T cells are unique to mammals and only found in extant marsupials and monotremes (47, 73). The *TRE* locus would represent the first example of the evolution of a novel TCR due to duplications of the *TRB* locus which, like *TRD* undergoes recombination of V, D, and J gene segments. Marsupials and monotremes also have conventional γδ T cells, consistent with TRG pairing with either TRD or TRM. If TRE pairs with TRA, as predicted, this would demonstrate that, like TRG, TRA can pair with multiple partners, TRB or TRE in this case. This would be consistent with the TCR loci that undergo V to J recombination having greater promiscuity in their pairing possibilities.

As noted above, the TCR locus duplications found so far have involved either *TRB* or *TRD* and not *TRA* or *TRG.* The *TRB* and *TRD* loci are rearranged first in developing αβ and γδ T cells, respectively. Although much of early γδ T cell development remains a mystery, much is known about αβ T cell development, notably the role the TCRβ chain plays as a developmental checkpoint (74). Having a second *TRB* or *TRB*-like locus that encodes chains that pair with TCRα may provide additional options for successful αβ T cell development. This may be particularly important for species dependent on αβ T cells due to lacking γδ T cells. In addition, TCR chains encoded by combinations of V, D, and J gene segments, such as *TRD* and *TRB*, typically have increased diversity. Such increased diversity may again provide an evolutionary advantage to species lacking T cell subsets.

Most transcripts encoded by the *TRE* locus found in two *T. rugosa* spleen transcriptome databases were non-functional. Nonetheless they contained evidence of having been transcribed from genes assembled by somatic V(D)J recombination. There is also evidence of *TRE* being transcribed in other squamate reptiles including in a transcriptome of the many-banded krait, *Bungarus multicinctus* (18). Similarly, the majority of *TRB* transcripts (58%) were also non-functional. It was surprising to find such a large percentage (86.4%) of non-functional transcripts for *TRE* in a *T. rugosa* peripheral lymphoid organ. Though there were more functional transcripts for *TRB* than *TRE* it appears common for TCRs to have fewer functional transcripts in *T. rugosa.* Whether this is due to poor selection in the thymus, development occurring outside the thymus, or nonsense-mediated decay of TCR transcripts remains unknown (75). The high percentage of non-functional transcripts does not appear to be common to all recombined immune genes, however, as most of the Ig transcripts for both heavy and light chains are productively rearranged (not shown, unpublished data).

It is also possible that the spleen is not the primary site of mature αϵ T cells in squamates. Indeed, αϵ T cells may be found in locations that are associated with γδ T cells, such as the skin, gut, or other epithelial sites (76–78). It is known that the thymus of certain reptiles including squamates can develop seasonally, however, how this affects the development of T cells, when αβ T cells develop in squamates, and their relationship to potential αϵ T cells is unknown (9, 79). Further research into the location of αϵ T cells, the timing of their development, their function, and their ligands is necessary.

## Conclusion

The lack of γδ T cells in squamates provides natural models with which to study evolutionary compensation to the wholesale loss of cell lineages in the adaptive immune system. Here, we confirm that the lack of γδ T cells has not resulted in increased genomic complexity of the genes encoding the potential αβTCR repertoire. Indeed, we have confirmed that αβTCR complexity is generally low in squamates compared to other amniote lineages. Noteworthy is duplication of the *TRB* locus giving rise to the *TRE* locus in squamates. *TRE* adds to the list of gene duplications giving rise to extra TCR loci not found in well-studied model species such as laboratory mice or humans. Whether αϵ T cells are compensating for the loss of γδ T cells in squamates is unknown. They do not appear to increase the potential overall diversity of T cells available to the host animal. The presence of functional or location differences between conventional αβ and the αϵ T cells remains to be determined.

## Data Availability

The datasets presented in this study can be found in online repositories. The names of the repository/repositories and accession number(s) can be found in the article/**Supplementary Material**.
