# Supplementary material for: Squamate reptiles may have compensated for the lack of γδTCR with a duplication of the TRB locus
Source: Front Immunol. 2025 Jan 9;15:1524471. doi: 10.3389/fimmu.2024.1524471 (PMC11754216; doi:10.3389/fimmu.2024.1524471)
Supplement: Supplementary file 1 [file DataSheet1.pdf]

Supplementary Figure 1

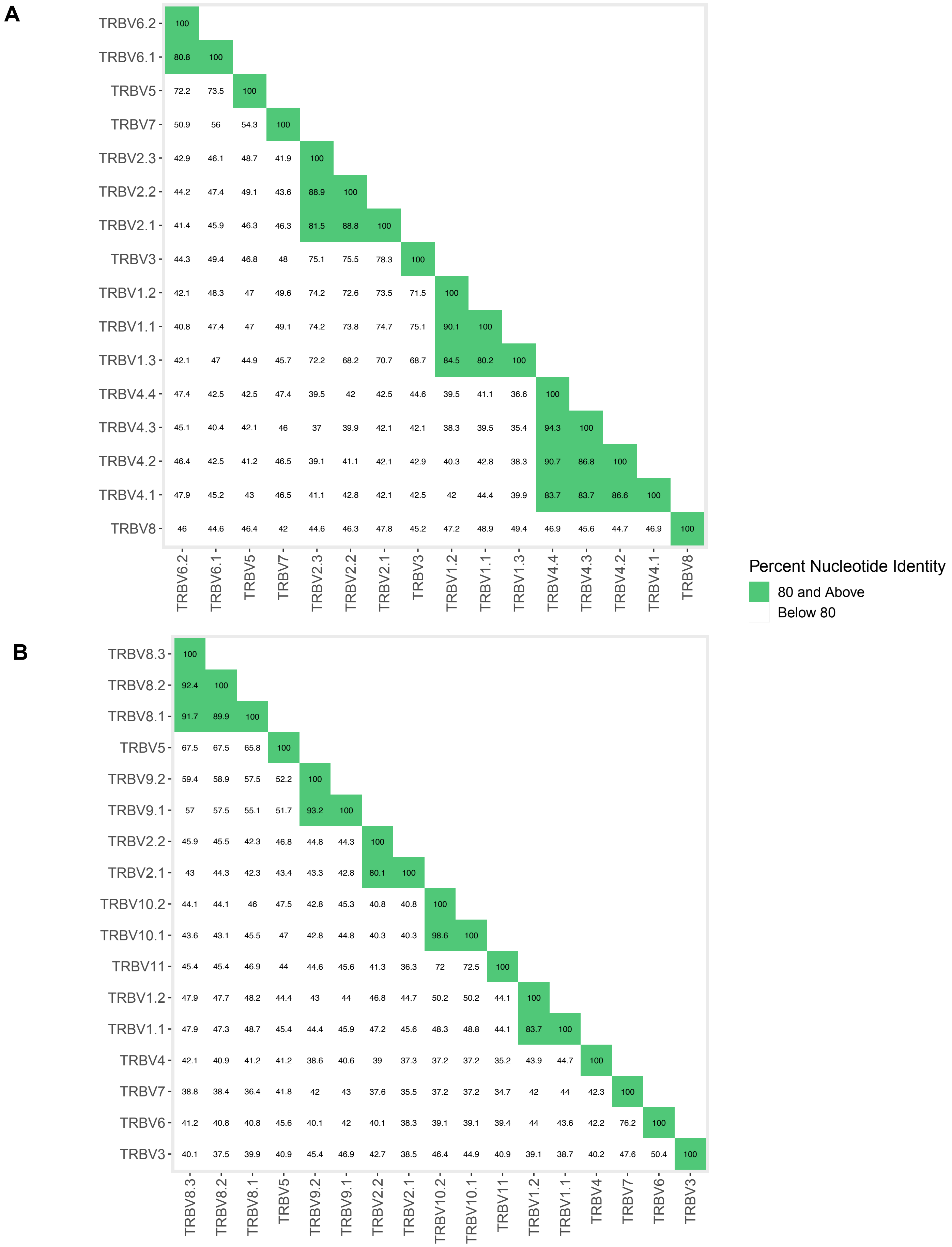

Supplementary Figure 1: *TRBV* nucleotide percent identity matrices. Matrices are based on nucleotide identity aligned with ClustalW. V families are based on ≥80% nucleotide identity. *TRBV* genes with ≥80% nucleotide identity are highlighted in green. **A.** Skink *TRBV* families. Vs shown are from haplotype 2. **B.** Tuatara *TRBV* families.
