# Supplementary material for: Squamate reptiles may have compensated for the lack of γδTCR with a duplication of the TRB locus
Source: Front Immunol. 2025 Jan 9;15:1524471. doi: 10.3389/fimmu.2024.1524471 (PMC11754216; doi:10.3389/fimmu.2024.1524471)
Supplement: Supplementary file 2 [file DataSheet2.pdf]

Supplementary Figure 2

A

```
                                           FR1    CDR1   FR2                 CDR2
                                                                                                                    FR3    CDR3    FR4
VB4.3             G ITVTQRDRFQIITVGSSTKIHCEHDDASYFTILWYRQERHGEKRQLQLIGISVQG   TDPEIEVKEYSIGRLSVKEASLSTPVQKAADSAVYFCAASKGLSGYEQTFGAGTKLTVLE P
VB7               WTDGKIHQPSSLVVEK-TTAQLE-QQN- NHDRMF--Q-QHNKGLQL- YHSLQVSL   AQR-ANSERFTAHRLKKTDFP-NISSVSLE--AT----S-RQD-SINLX--T-NR-LLX- N
VB8               -SPAIF-KPQSVVESP-GRATLD-FLEES-NPNFY----RPGGQL  E QLTFSIST  GMSQNLGPEHIKGERPKDNTFT-RLTTLLAN-TGT-Y--W-HEDNGDAH---E-------- P
VB1.2             SGYAM KQPPYMVVEK-KPETLT-SQLKTQHNNMY--K-ERENSPKLH LVIYSIEGAGGKVEDEFQNHFLSSGTKNYELS-QLKSARLE--GT----KQDRRYNAPLS--E--R-A--- P
                        ::    :   *    : *   . .   : **:*.             .:.       :   :.      .     *        *:..*:** .    .    ** *.:* : *
```

B

```
                                FR1       CDR1    FR2                   CDR2
                                                                                                                   FR3     CDR3       FR4
SAMAR4_VE1        TQQPAFARFSQQSKHTFSCSVS  GTDYNIFWYRQFPGTTELQVLGILYFSDDKLKDTLDKPNSKNWLSGKWVKKGVSMNLELKDLQPDDTGLYLCGASIQQ RA QMYFXGAGHAGPRR N
WA2_VE1           ------A-F----KHTFS----ASSTD-----------------------------------D-----------------------------------NGYGAGK PDVLR--------- P
SAMAR1_VE2        ----P-V-KP-TGTMELN-T-D  QDN-DFY----I--Q----Q--VYKAFGNS-DDKQKGLET  R-  SSKRN-KQ-F-T-T---SQ--------SRIGTPD    VLR--------- P
WA1_VE2           ----P-V-KP-TGTMELN-T-D  QDN-DFY----I--Q----Q--VYKAFGNS-DDKRKGLET  R-  SSKRN-KQ-F-T-T---SQ--------SKIQGTTATRCTSX--------- N
SAMAR2_VE2        ----P-V-KP-TGTMELN-T-D  QDN-DFY----I--Q----Q--VYKAFGNS-DDKRKGLET  R-  SSKRN-KQ-F-T-T---SQ--------NSAY Q    MYFX--------- N
SAMAR3_VE2        ----P-V-KP-TGTMELN-T-D  QDN-DFY----I--Q----Q--VYKAFGNS-DDKQKGLET  R-  SSKRN-KQ-F-T-T---SQ--------SKDTAP    DVLR--------- P
                  **** *.*  * ..  :.*:*.    :*:::****:** **** **:    .:.*.*. .  ::   *  .  ::* .* * *.*** :********              *********
```

Supplementary Figure 2: Alignments of V sequences from spleen transcriptome(s). Sequences were aligned with ClustalW. Gaps are represented by spaces. Sequences identical to the top sequence are represented by dashes. Productive (P) and nonproductive (N) rearrangements are labeled on the side. Framework regions (FR) and complementarity determining regions (CDR) are labeled above the alignments. Vs are labeled based on family. Red X's represent inserts/deletions. Asterisks represent identical amino acids in all sequences. Colons represent amino acid substitutions with similar R groups. Periods represent semi-conserved amino acid substitutions. **A.** An alignment of *TRBV* sequences from the South Australian skink spleen transcriptome. V sequences shown represent Vs that were present in the transcriptomes. Duplicate sequences were not included. Inverted *TRBV* underlined. **B.** An alignment of *TREV* sequences from both the South Australian (SAMAR) and West Australian (WA) skink spleen transcriptomes.
