# Supplementary material for: Squamate reptiles may have compensated for the lack of γδTCR with a duplication of the TRB locus
Source: Front Immunol. 2025 Jan 9;15:1524471. doi: 10.3389/fimmu.2024.1524471 (PMC11754216; doi:10.3389/fimmu.2024.1524471)
Supplement: Supplementary file 3 [file DataSheet3.pdf]

Supplementary Figure 3

A

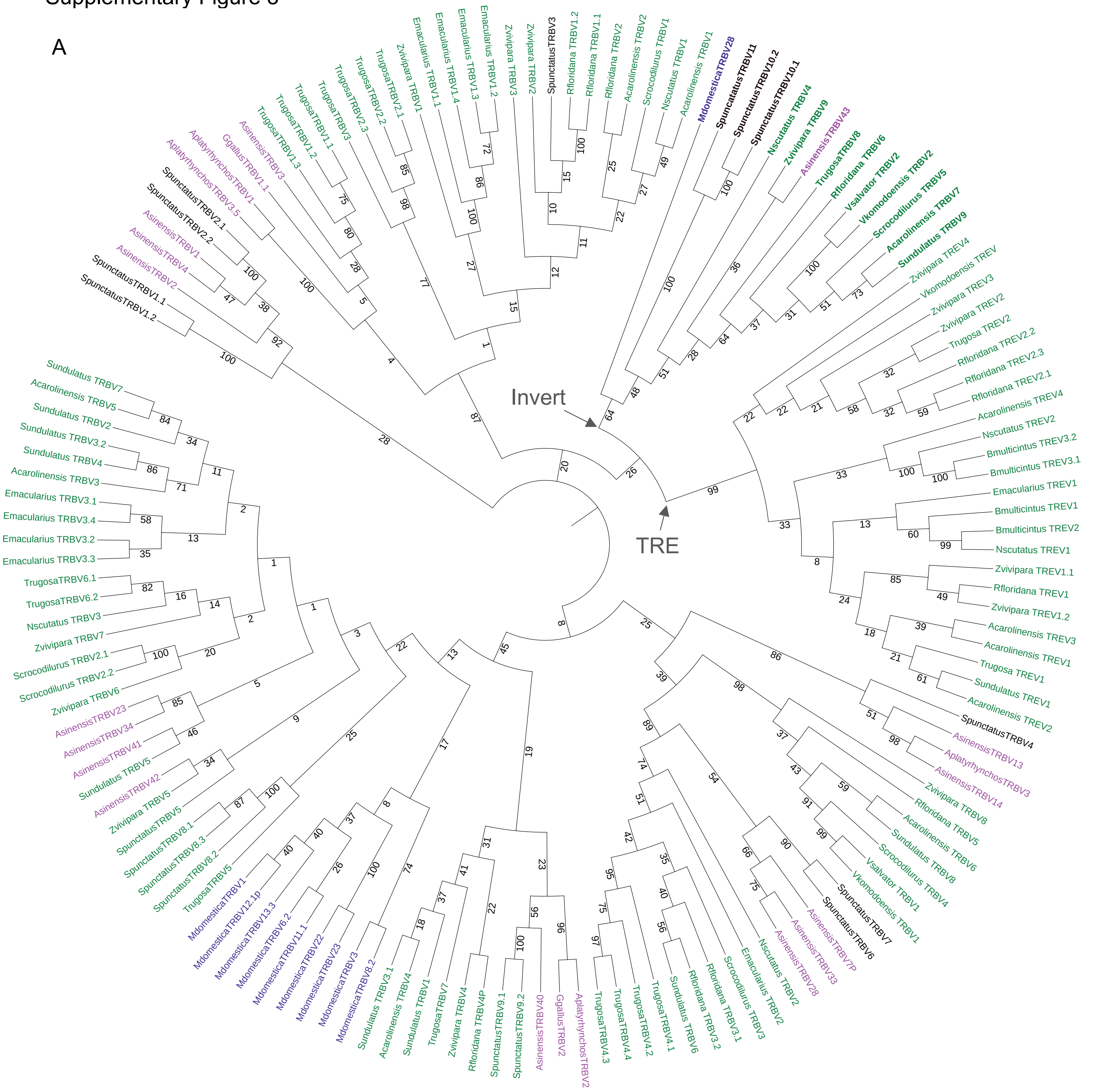

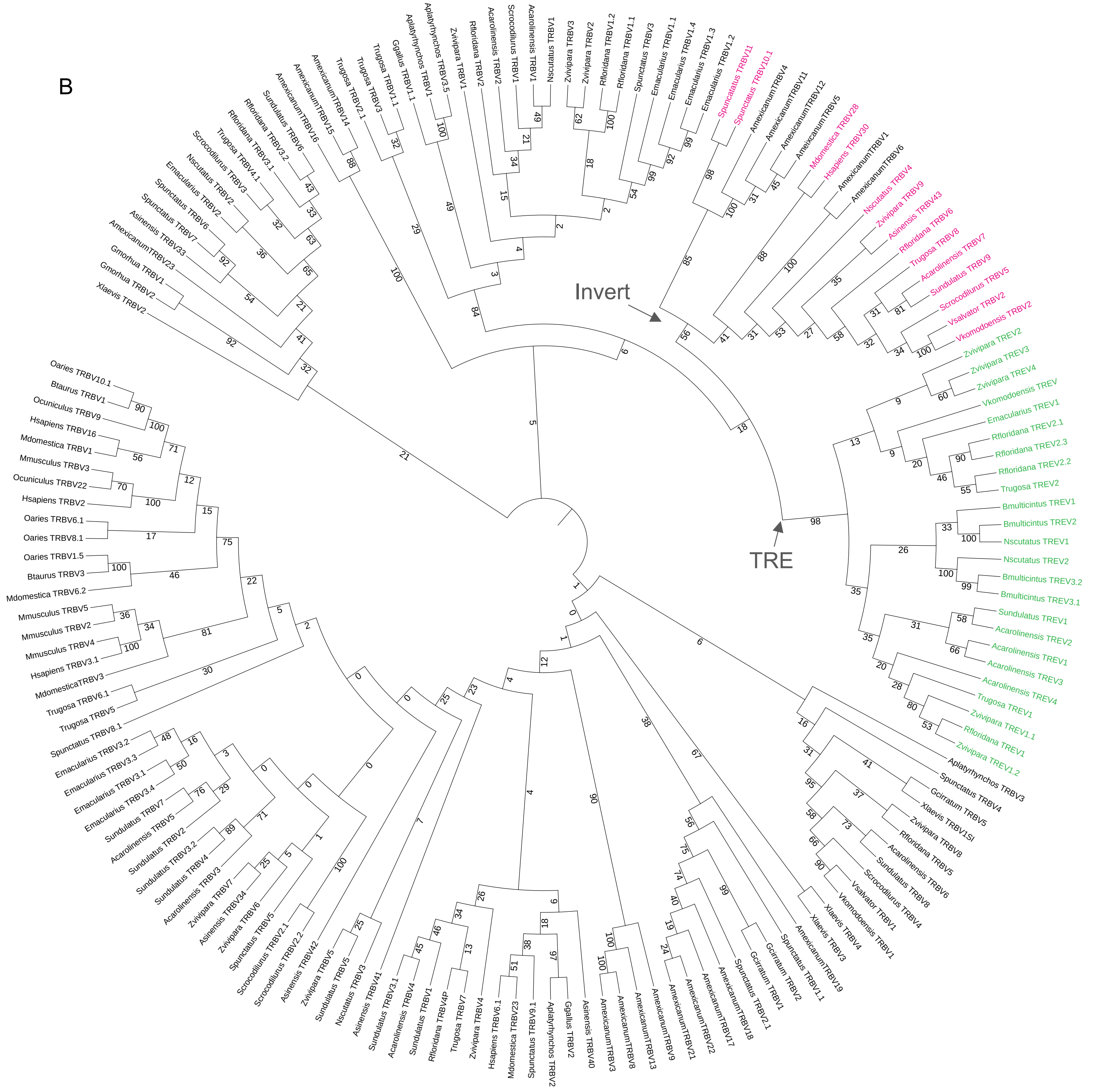

Supplementary Figure 3: Neighbor-joining trees based on an amino acid alignment of vertebrate *TRBV* and *TREV* genes. Numbers on branches indicate bootstrap values on 1,000 replicates. Similar results were found in multiple iterations of the trees including minimum evolution and maximum likelihood. The clade containing the inverted *TRBV*s is bolded and labeled with an arrow and “Invert”. *TREVs* are also indicated by arrow and labeled “TRE”. **A.**

Tree containing *TRBV* and *TREV* from 1 representative mammal (blue), 11 squamate reptiles (green), one Rhynchocephalian (black), and 3 archelosaurs (purple) and demonstrating that the *TRBV* families of squamates intersperse amongst the tree, and *TREVs* are most related to the 3'-inverted *TRBV*. **B.** Tree containing *TRBV* and *TREV* from 6 mammals, 11 squamate reptiles, 1 Rhynchocephalian, 3 archelosaurs, 2 amphibians, 1 teleost fish, and 1 cartilaginous fish. *TREVs* cluster with 3'-inverted amniote *TRBV* sequences. 3'-inverted amniote *TRBV* (pink) and squamate *TREV* (green) are highlighted. Mammals used in the trees include short-tailed opossum (*M. domestica*), human (*H. sapiens*), mouse (*M. musculus*), cow (*B. taurus*), sheep (*O. aries*), and rabbit (*O. cuniculus*); squamates include skink (*T. rugosa*), common lizard (*Z. vivipara*), Komodo dragon (*V. komodoensis*), water monitor (*V. salvator*), Florida worm lizard (*R. floridana*), mainland tiger snake (*N. scutatus*), many-banded krait (*B. multicintus*), anole (*A. carolinensis*), leopard gecko (*E. macularius*), Chinese crocodile lizard (*S. crocodilurus*), and fence lizard (*S. undulatus*); the Rhynchocephalian is tuatara (*S. punctatus*); the archelosaurs include Chinese alligator (*A. sinensis*), chicken (*G. gallus*), and duck (*A. platyrhynchos*); the amphibians include axolotl (*A. mexicanum*), African clawed from (*X. laevis*), and Western clawed frog (*X. tropicalis*). The teleost is cod (*G. morhua*); the cartilaginous fish is nurse shark (*G. cirratum*). Accession numbers of sequences used in the trees can be found in Supplementary Table 5.
