# Supplementary material for: Squamate reptiles may have compensated for the lack of γδTCR with a duplication of the TRB locus
Source: Front Immunol. 2025 Jan 9;15:1524471. doi: 10.3389/fimmu.2024.1524471 (PMC11754216; doi:10.3389/fimmu.2024.1524471)
Supplement: Supplementary file 4 [file DataSheet4.pdf]

Supplementary Figure 4

```
Trugosa_TREC                   LLGP   ACHNGRVVTLVCVAQDFPYAWPLDVHWESAAGEELPRLAVA
Trugosa_TRBC       ESEDDISPPTVTIFDPSAQELQEKQKVTIVCLVTDFYP DHVNLTWSVDGDERT KGVKT
Ggallus_TRBC        KNSEIIEPDVVIFSPSKQEIQEKKKATLVCLASGFFP DHLNLVWKVNGVKRT EGVGT
Spunctatus_TRBC     DDYEITPPRVAIFSPSKQEIKEKKKATLVCLARGFYP DHVNLTWWVNGARRT EGVKT
Spunctatus_TRGC      NLQ       ILPP   SPVQEGKETYICFFENFYP EVVKATWREGDKRDKVLDS V
Spunctatus_TRDC                          NDGQYTAACLAKDFYP KNIEITIKPGDNLYDLKDA I
Ggallus_TRAC                                 NLEMCLITDYSP EKLDLSSVDSKTETVVEVA T
Trugosa_TRAC         NLQKSEPS  VYEL   ESTNKSHPLSACLFTDYFP GNINVSATGSNLVGI ESS V
                                                 *.   .:      :.

Trugosa_TREC       TEPVQPMGGSCSFSTVSRLSMPATQWQKS     R GYSCHVRQGDAPLVSAH
Trugosa_TRBC       KEPD-LDEAAKKFSLISRLRITRKEWEKT     K EIQCNVYFDPDKR  NYTGT
Ggallus_TRBC       DEISTS  NGSTYSLTSRLRISAQEWFNP     LNRFECIANFFKNGTQQSIQKI
Spunctatus_TRBC    DDYSKRDDKAKSYSLTSRLRITDQEWFNS     RNHFKCHVDFHGDE IQTFDDA
Spunctatus_TRGC    QAD AFKFNETSYSA        SSWMTVDASDLSKKYTCEIQHEAGSN  TIEFLPAGP
Spunctatus_TRDC    LSP    DGTYSSVK        VVKVSP     GQQVHCSALHDGIHI NATEAQQATI
Ggallus_TRAC       SEN    KHEASYLS        TYWAKK      DEMQCGAKHEGFGI LKGDDPEAGA
Trugosa_TRAC       VVV    EDQASYGA        VLWQNA     NENLQCIANYDGEDI QAKKDI

Trugosa_TREC                 LGGSDARLQEGPSEMQLNLRTGQLIFLLLTIKSFAYGAVLAAYTTCRKIG
Trugosa_TRBC                IRGQVCDGSDALKEPYLRSNNLGKLIYILLIFKSTLYGAFITGLMLRKKSM
Ggallus_TRBC                IY  GDTGCDIFKENYQRSATAGKFVYIMLIFKSILYGIFVMGMMLWYKKM
Spunctatus_TRBC             IN  GAEGCSITPESYLRQTNATKFTYRMLLFKALLYALLVSALM-WKAKT
Spunctatus_TRGC    EN QDTPDCIPTPEDSTAVSEDIGGRFTHNTALLVYVVLLLKSTIYYIIILFLV YRMGG
Spunctatus_TRDC    QPLTVKQEYCPPANTTVPEDESTEKVNTVSVIILGLRVLFAKSIAFNVLMTTKL MFF
Ggallus_TRAC       ST     VCITGMS   LLFKTDENLNMLTFSQLGLKIIFMKAVIFNVLITMLM WKKNQ
Trugosa_TRAC        A     DSCSGTQ   MSFQTDERLNLLSLTVLGLRVIFFKSVAVNLLLTFRL WSR
                                                  :   ::  *:      .:

Trugosa_TREC       L
Trugosa_TRBC       KEKPFA
Ggallus_TRBC       Y
Spunctatus_TRBC    GDKLSRE
Spunctatus_TRGC    LIKPQSKKP
Spunctatus_TRDC
Ggallus_TRAC
Trugosa_TRAC
```

**Supplementary Figure 4:** An alignment of T cell receptor constant regions. Species include the chicken (*Gallus gallus*), the tuatara (*Sphenodon punctatus*), and the skink (*Tiliqua rugosa*). Constant regions were aligned with ClustalW. Transmembrane regions were identified with DeepTMHMM-2.0. Transmembrane regions are unlined. Conserved residues in the transmembrane region are bolded. TCRα and TCRδ contain the conserved Arg and Lys. However, in the chicken the first conserved Arg is a Lys. TCRβ, TCRγ, and TCRε all contain the single Lys.
